# Endospores of halophilic bacteria of the family *Bacillaceae *isolated from non-saline Japanese soil may be transported by Kosa event (Asian dust storm)

**DOI:** 10.1186/1746-1448-1-8

**Published:** 2005-10-20

**Authors:** Akinobu Echigo, Miki Hino, Tadamasa Fukushima, Toru Mizuki, Masahiro Kamekura, Ron Usami

**Affiliations:** 1 Department of Applied Chemistry, Faculty of Engineering, Toyo University, 2100 Kujirai, Kawagoe, Saitama 350-8585, Japan; 2 Bio-Nano Electronics Research Centre, Toyo University, 2100 Kujirai, Kawagoe, Saitama 350-8585, Japan; 3 Noda Institute for Scientific Research, 399 Noda, Noda, Chiba 278-0037, Japan

## Abstract

**Background:**

Generally, extremophiles have been deemed to survive in the extreme environments to which they had adapted to grow. Recently many extremophiles have been isolated from places where they are not expected to grow. Alkaliphilic microorganisms have been isolated from acidic soil samples with pH 4.0, and thermophiles have been isolated from samples of low temperature. Numerous moderately halophilic microorganisms, defined as those that grow optimally in media containing 0.5–2.5 Molar (3–15%) NaCl, and halotolerant microorganisms that are able to grow in media without added NaCl and in the presence of high NaCl have been isolated from saline environments such as salterns, salt lakes and sea sands. It has tacitly been believed that habitats of halophiles able to grow in media containing more than 20% (3.4 M) are restricted to saline environments, and no reports have been published on the isolation of halophiles from ordinary garden soil samples.

**Results:**

We demonstrated that many halophilic bacteria that are able to grow in the presence of 20% NaCl are inhabiting in non-saline environments such as ordinary garden soils, yards, fields and roadways in an area surrounding Tokyo, Japan. Analyses of partial 16S rRNA gene sequences of 176 isolates suggested that they were halophiles belonging to genera of the family *Bacillaceae*, *Bacillus *(11 isolates), *Filobacillus *(19 isolates), *Gracilibacillus *(6 isolates), *Halobacillus *(102 isolates), *Lentibacillus *(1 isolate), *Paraliobacillus *(5 isolates) and *Virgibacillus *(17 isolates). Sequences of 15 isolates showed similarities less than 92%, suggesting that they may represent novel taxa within the family *Bacillaceae*.

**Conclusion:**

The numbers of total bacteria of inland soil samples were in a range from 1.4 × 10^7^/g to 1.1 × 10^6^/g. One tenth of the total bacteria was occupied by endospore-forming bacteria. Only very few of the endospore-forming bacteria, roughly 1 out of 20,000, are halophilic bacteria. Most of the halophilic bacteria were surviving as endospores in the soil samples, in a range of less than 1 to about 500/g soil. Samples collected from seashore in a city confronting Tokyo Bay gave the total numbers of bacteria and endospores roughly 1000 time smaller than those of inland soil samples. Numbers of halophilic bacteria per gram, however, were almost the same as those of inland soil samples. A possible source of the halophilic endospore originating from Asian dust storms is discussed.

## Background

Extremophiles are microorganisms adapted to grow in conditions such as extreme pH, temperature, salinity and absence of oxygen [1]. The representatives are acidophiles (*Thiobacillus ferroxidans*), alkaliphiles (*Bacillus alcalophilus*), hyperthermophiles (*Thermoproteus tenax*), extreme halophiles (*Halobacterium salinarum*) and methanogens (*Methanobacterium formicicum*). In general, it has been believed that they survive in the extreme environments to which they had adapted to grow. Many extremophiles, however, have been isolated from places where they are not expected to grow. Alkaliphilic microorganisms were isolated from acidic soil samples with pH 4.0 as well as from neutral and alkaline soil [2]. Thermophiles have been isolated from environments of high temperature and also from samples of lower temperature such as soil, food, animal's excrement and seawater [3]. For example, *Bacillus stearothermophilus *(now *Geobacillus stearothermophilus*) and *Clostridium thermoautotrophicus *(now *Moorella thermoautotrophica*) were isolated from ordinary soil. Strictly anaerobic bacteria such as methanogens, sulfate-reducers, and homoacetogens were isolated from rice paddies during dry fallow period, arable soils, and even from desert soils [4,5]. Thus, the notion that isolation of an organism from a given environment does not mean that the organism is growing in that environments, but just surviving is now generally accepted.

Halophilic microorganisms are adapted to conditions of high salinity and require a certain concentration of NaCl for their optimum growth [6,7]. They have been isolated from various saline environments such as salt lakes (eg. the Dead Sea, the Great Salt Lake), salterns, solar salts and subsurface salt formation. Extremely halophilic microorganisms require high concentration of NaCl for their growth, with optimum concentrations of 2.5–5.2 M (15–30%). *Haloarcula vallismortis *and *Haloterrigena turkmenica *for example, have been isolated from salt pool of Death Valley, California, and saline soil of Turkmenia, respectively [8,9]. Moderate halophiles are defined as those that grow optimally in media containing 0.5–2.5 M (3–15%) NaCl, such as *Halomonas maura *isolated from a saltern in Morocco, and *Marinococcus halophilus *isolated from sea sands [10,11]. Halotolerant microorganisms possess the ability to grow in media without added NaCl and also in the presence of high concentrations of NaCl. For example, *Halobacillus salinus *isolated from a salt lake in Korea is able to grow without added salt and in media containing up to 23% NaCl [12].

Are halophiles inhabiting non-saline environments such as garden soil, yards and field? *Bacillus clarkii*, *B. agaradhaerens *and *B. pseudofirmus *are examples of halotolerant bacteria isolated from soil samples that were shown to be tolerant up to 16% or 17% NaCl [13]. It has, however, been tacitly believed that habitats of halophiles able to grow in media containing higher concentrations, let's say 20% (3.4 M), are restricted to saline environments [14,15], and no reports have been published on the isolation of microorganisms able to grow at 20% or higher NaCl concentrations from ordinary, non-saline soil samples. In 1980 Onishi et al. [14] surveyed extensively the occurrence of halophilic bacteria in more or less saline samples collected in Japan. They adopted enrichment culture in a medium containing 4 M (23.4%) NaCl, a customary concentration for the cultivation of *Halobacterium *spp. They isolated 168 strains finally, but no enrichment was obtained from one third of 287 samples of sea sands and seaweeds collected on seashore. They did not include ordinary garden soil samples. It should be pointed out that a non-pigmented haloarchaeon strain 172P1 (designated later as *Natrialba asiatica *[16]) was isolated during their survey from dry beach sands with granular salts attached.

In this study, we defined "halophilic bacteria", for convenience, as microorganisms that form colonies on agar plates of a complex medium with 20% added NaCl, and demonstrated that halophilic bacteria are inhabiting in non-saline environments such as ordinary garden soils, yards, fields and roadways in an area surrounding Tokyo, Japan. Phylogenetic analyses of the isolates suggested that they were halophiles belonging to genera of the family *Bacillaceae*.

## Results

### Isolation of halophilic bacteria from soil samples

**Figure 1:**
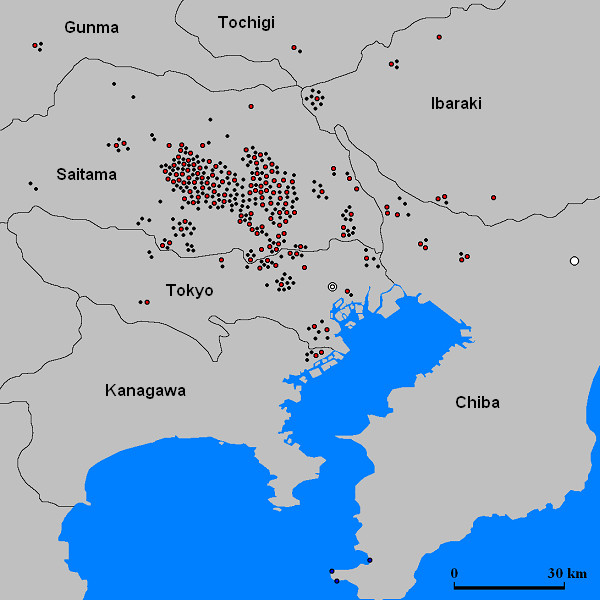
**Collection sites of the 360 soil samples**. Red circles; colonies were detected from at least one of the three plates of different pH, black circles; colonies were not detected. A white double circle indicates Tokyo Station, and a white circle indicates Narita Airport.

Soil samples (0.5 g each) taken from 360 places were spread on agar plates containing 20% NaCl, with pH adjusted to 5.0, 7.0 and 9.0 respectively. The pH of the soil samples ranged between 5.0 and 6.0. After incubation of plates for 3 weeks at 37°C, colony formations were observed in 132 soil samples (red circles in Fig. 1), at least on one of the three agar plates. Numbers of colonies per plate ranged from 1 on 51 plates to 40 on 1 plate. The sum of colonies amounted to 49 from the medium of pH 9.0, 534 from the pH 7.0 medium, and 61 from the medium of pH 5.0. By inspecting each plate, representative colonies were picked up and transferred to fresh plates and purified by plating out of serial dilutions. A few isolates gradually failed to form colonies on fresh plates. When colonies failed to grow on fresh 20% NaCl plates, concentration of NaCl was decreased to 15 or 10%. It was observed that 26 strains failed to grow in the presence of 20% NaCl. According to our definition of the present paper, these strains are not 'halophilic bacteria', but these strains were included in the further characterization (see discussion).

Finally, 176 strains were obtained (Table 1): 27 strains from 23 samples on alkaline medium (pH 9.0), 139 strains from 120 samples on neutral medium (pH 7.0), and 10 strains from 9 samples on acidic medium (pH 5.0). Endospores were observed by microscope after spore staining [17]. These strains have been kept at 5°C on agar plates of 10% NaCl.

On the other hand, from 228 soil samples (black circles in Fig. 1), about two thirds of the 360 samples collected, no colonies appeared on any plates of the three different pH values. There was no distinct bias in the distribution of black and red circles. In order to estimate if those soil samples contain indeed no microorganisms able to grow at 20% NaCl, two soil samples were randomly picked up, and 0.5 g each was spread on to 10 agar plates of pH 7.0. The colony numbers per plate ranged from 0 on 3 plates to 5 in 1 plate, amounting to 14 in sample 1. From another sample the numbers were from 0 on 3 plates to 4 on 1 plate, amounting to 14 colonies. These data may suggest that halophilic bacteria able to grow at 20% NaCl inhabit any soil samples, at least at a frequency of 1 c.f.u. (colony forming unit)/g soil, in the area we investigated.

**Table 1:** Strains isolated from ordinary soil samples on agar plates containing 20% NaCl.

Strain No.	Sampling site	Pig.	NaCl (M)		pH		Similarity	Tentatively assigned to
			
			Range	Optimum	Range	Optimum	(%)	
3	Omiya, S	-	0.9–2.6	0.9–1.7	6.5–10.0	8.5–9.5	100	*B. haloalkaliphilus *(AJ238041)
12	Showa, S	-	0.9–4.3	1.7–2.6	6.5–10.0	8.5–9.5	100	*B. haloalkaliphilus*
27	Takasaki, G	-	1.7–3.4	1.7–2.6	6.5–10.0	8.5–9.5	100	*B. haloalkaliphilus*
29	Kamagaya, C	-	1.7–4.3	1.7–2.6	6.5–10.0	8.5–9.5	100	*B. haloalkaliphilus*
1	Wako, S	-	0.9–4.3	1.7–2.6	6.5–10.0	8.5–9.5	99.8	*B. haloalkaliphilus*
28	Sakado, S	-	1.7–2.6	1.7–2.6	6.5–10.0	8.5–9.5	99.8	*B. haloalkaliphilus*
7	Higashichichibu, S	-	0.9–1.7	0.9–1.7	6.5–10.0	8.5–9.5	99.6	*B. haloalkaliphilus*
8	Higashichichibu, S	-	0.9–2.6	0.9–1.7	6.5–10.0	8.5–9.5	99.6	*B. haloalkaliphilus*
2	Yachiyo, C	-	0–3.4	0.9–1.7	6.5–10.0	8.5–9.5	98.4	*B. haloalkaliphilus*
18	Kawagoe, S	-	0.9–4.3	1.7–2.6	6.5–10.0	8.5–9.5	98.0	*B. haloalkaliphilus*
31	Okabe, S	-	0–3.4	1.7–2.6	6.5–10.0	6.5–7.5	97.2	*F. milosensis *(AJ238042)
19	Wako, S	-	0.9–4.3	1.7–2.6	6.5–10.0	8.5–9.5	94.0	*F. milosensis*
9	Shiki, S	-	0–3.4	0.9–1.7	6.5–10.0	6.5–7.5	96.1	*G. halotolerans *(AB101591)
17	Urawa, S	-	0–4.3	0.9–1.7	6.5–10.0	8.5–9.5	91.4	*B. agaradhaerens *(X76445)
22	Kawagoe, S	-	1.7–4.3	1.7–2.6	6.5–10.0	8.5–9.5	88.8	*H. trueperi *(AJ310149)
14	Kawagoe, S	Y	0.9–3.4	1.7–2.6	6.5–10.0	8.5–9.5	88.7	*H. trueperi*
13	Kawagoe, S	Y	0.9–3.4	1.7–2.6	6.5–10.0	8.5–9.5	88.2	*H. trueperi*
25	Okegawa, S	-	1.7–3.4	1.7–2.6	6.5–10.0	8.5–9.5	88.0	*H. trueperi*
10	Tsurugashima, S	B	1.7–2.6	1.7–2.6	6.5–10.0	8.5–9.5	88.0	*'B. nitritophilus' *(AJ309562)
11	Showa, S	Y	0–3.4	1.7–2.6	6.5–10.0	8.5–9.5	87.8	*'B. nitritophilus'*
16	Iruma, S	B	1.7–3.4	1.7–2.6	6.5–10.0	8.5–9.5	87.7	*'B. nitritophilus'*
4	Matsubushi, S	-	1.7–4.3	1.7–2.6	6.5–10.0	8.5–9.5	87.5	*'B. nitritophilus'*
21	Omiya, S	B	1.7–4.3	1.7–2.6	6.5–10.0	8.5–9.5	87.5	*'B. nitritophilus'*
15	Omiya, S	B	1.7–3.4	1.7–2.6	6.5–10.0	8.5–9.5	87.3	*'B. nitritophilus'*
5	Kawasaki, K	B	1.7–4.3	1.7–2.6	6.5–10.0	8.5–9.5	86.9	*'B. nitritophilus'*
24	Soka, S	-	0–4.3	0.9–1.7	6.5–10.0	8.5–9.5	86.9	*'B. nitritophilus'*
30	Okabe, S	-	1.7–4.3	1.7–2.6	6.5–10.0	8.5–9.5	87.3	*'Pc. psychrotoleratus' *(AF324659)
								
61	Omiya, S	-	0–3.4	0–0.9	6.5–8.0	6.5–7.5	97.3	*F. milosensis *(AJ238042)
66	Koga, I	-	0–2.6	0–0.9	6.5–8.0	6.5–7.5	97.1	*F. milosensis*
173	Tsurugashima, S	-	0–3.4	0–0.9	6.5–8.0	6.5–7.5	97.1	*F. milosensis*
106	Showa, S	-	0–3.4	0–0.9	6.5–8.0	6.5–7.5	97.0	*F. milosensis*
112	Yachiyo, C	-	0–3.4	0.9–1.7	6.5–8.0	6.5–7.5	97.0	*F. milosensis*
113	Yachiyo, C	Y	0–3.4	0–0.9	6.5–8.0	6.5–7.5	97.0	*F. milosensis*
117	Niiza, S	-	0–3.4	0–0.9	6.5–8.0	6.5–7.5	97.0	*F. milosensis*
168	Tsurugashima, S	-	0–3.4	0–0.9	6.5–8.0	6.5–7.5	97.0	*F. milosensis*
176	Kawagoe, S	-	0–3.4	0–0.9	6.5–8.0	6.5–7.5	97.0	*F. milosensis*
70	Okegawa, S	-	0–3.4	0–0.9	6.5–8.0	6.5–7.5	96.9	*F. milosensis*
105	Kashiwa, C	-	0–3.4	0–0.9	6.5–8.0	6.5–7.5	96.9	*F. milosensis*
116	Niiza, S	Y	0–3.4	0–0.9	6.5–8.0	6.5–7.5	96.9	*F. milosensis*
172	Ageo, S	-	0–3.4	0–0.9	6.5–8.0	6.5–7.5	96.9	*F. milosensis*
69	Kitamoto, S	-	0–3.4	0–0.9	6.5–8.0	6.5–7.5	96.7	*F. milosensis*
170	Asaka, S	-	0–3.4	0–0.9	6.5–8.0	6.5–7.5	96.7	*F. milosensis*
60	Kasukabe, S	-	0–3.4	0–0.9	6.5–8.0	6.5–7.5	96.5	*F. milosensis*
185	Urawa, S	-	0–3.4	0–0.9	6.5–8.0	6.5–7.5	96.4	*F. milosensis*
74	Hanyu, S	-	0–3.4	0–0.9	5.5–8.0	6.5–7.5	96.3	*G. halotolerans *(AB101591)
75	Hanyu, S	-	0–2.6	0–0.9	6.5–8.0	6.5–7.5	96.3	*G. halotolerans*
76	Katsushika, T	-	0–3.4	0–0.9	6.5–8.0	6.5–7.5	96.3	*G. halotolerans*
102	Omiya, S	-	0–2.6	0–0.9	5.5–8.0	6.5–7.5	96.3	*G. halotolerans*
72	Kawagoe, S	-	0–2.6	0–0.9	6.5–8.0	6.5–7.5	96.1	*G. halotolerans*
77	Katsushika, T	-	0–3.4	1.7–2.6	6.5–8.0	6.5–7.5	94.8	*H. karajensis *(AJ486874)
51	Okegawa, S	-	0–3.4	1.7–2.6	6.5–8.0	6.5–7.5	100	*H. litoralis *(X94558)
54	Fujimi, S	-	0–3.4	0.9–2.6	6.5–8.0	6.5–7.5	100	*H. litoralis*
92	Fujimi, S	Y	0.9–2.6	0.9–1.7	6.5–8.0	6.5–7.5	100	*H. litoralis*
188	Ageo, S	-	0–3.4	0–0.9	6.5–8.0	6.5–7.5	100	*H. litoralis*
195	Fujimi, S	-	0–4.3	0–0.9	6.5–8.0	6.5–7.5	99.5	*H. litoralis*
200	Kawagoe, S	-	0–3.4	0–0.9	6.5–8.0	6.5–7.5	99.4	*H. litoralis*
91	Itabashi, T	-	0.9–3.4	0.9–2.6	6.5–8.0	6.5–7.5	98.8	*H. litoralis*
130	Sakado, S	-	0.9–2.6	0.9–1.7	6.5–8.0	6.5–7.5	98.8	*H. litoralis*
196	Urawa, S	Y	0–3.4	0.9–1.7	5.5–8.0	6.5–7.5	98.6	*H. litoralis*
152	Kawagoe, S	-	0–3.4	0.9–1.7	6.5–8.0	6.5–7.5	97.2	*H. litoralis*
136	Urawa, S	-	0–3.4	1.7–2.6	6.5–8.0	6.5–7.5	97.0	*H. litoralis*
97	Higashichichibu, S	-	0–3.4	0–0.9	5.5–8.0	6.5–7.5	96.7	*H. litoralis*
154	Ina, S	-	0–3.4	0.9–1.7	6.5–8.0	6.5–7.5	96.6	*H. litoralis*
78	Shiki, S	-	0–3.4	0.9–1.7	6.5–8.0	6.5–7.5	96.4	*H. litoralis*
132	Urawa, S	-	0–3.4	0.9–1.7	6.5–8.0	6.5–7.5	96.4	*H. litoralis*
180	Toda, S	-	0.9–3.4	0.9–1.7	6.5–8.0	6.5–7.5	96.4	*H. litoralis*
107	Showa, S	Y	0–3.4	0.9–2.6	6.5–8.0	6.5–7.5	96.3	*H. litoralis*
143	Nerima, T	-	0–3.4	0.9–1.7	5.5–8.0	6.5–7.5	96.3	*H. litoralis*
162	Kawagoe, S	-	0–3.4	0.9–1.7	6.5–8.0	6.5–7.5	96.3	*H. litoralis*
86	Wako, S	-	0–2.6	0.9–2.6	6.5–8.0	6.5–7.5	96.2	*H. litoralis*
88	Fujimi, S	-	0–3.4	0.9–2.6	6.5–8.0	6.5–7.5	96.2	*H. litoralis*
119	Toride, I	-	0.9–3.4	0.9–1.7	6.5–8.0	6.5–7.5	96.2	*H. litoralis*
131	Iruma, S	-	0–3.4	0–0.9	6.5–8.0	6.5–7.5	96.2	*H. litoralis*
133	Urawa, S	-	0.9–3.4	0.9–1.7	6.5–8.0	6.5–7.5	96.2	*H. litoralis*
164	Ota, T	-	0.9–3.4	0.9–1.7	6.5–8.0	6.5–7.5	96.2	*H. litoralis*
192	Tama, T	-	0–4.3	0.9–1.7	6.5–8.0	6.5–7.5	96.2	*H. litoralis*
55	Wako, S	-	0–3.4	0.9–2.6	6.5–8.0	6.5–7.5	96.1	*H. litoralis*
68	Okegawa, S	-	0–3.4	1.7–2.6	6.5–8.0	6.5–7.5	96.1	*H. litoralis*
84	Higashimurayama, T	Y	0–3.4	0.9–2.6	6.5–8.0	6.5–7.5	96.1	*H. litoralis*
85	Toshima, T	-	0–3.4	0.9–2.6	6.5–8.0	6.5–7.5	96.1	*H. litoralis*
95	Soka, S	-	0–3.4	0.9–1.7	6.5–8.0	6.5–7.5	96.1	*H. litoralis*
100	Higashichichibu, S	-	0–3.4	0.9–2.6	5.5–8.0	6.5–7.5	96.1	*H. litoralis*
104	Omiya, S	Y	0–3.4	0.9–1.7	6.5–8.0	6.5–7.5	96.1	*H. litoralis*
124	Koto, T	-	0–3.4	0–0.9	6.5–8.0	6.5–7.5	96.1	*H. litoralis*
144	Sakado, S	-	0–3.4	0–0.9	6.5–8.0	6.5–7.5	96.1	*H. litoralis*
145	Sakado, S	-	0–4.3	0.9–1.7	6.5–8.0	6.5–7.5	96.1	*H. litoralis*
151	Tsurugashima, S	-	0–3.4	0.9–1.7	6.5–8.0	6.5–7.5	96.1	*H. litoralis*
184	Yoshimi, S	Y	0–3.4	0.9–1.7	6.5–8.0	6.5–7.5	96.1	*H. litoralis*
203	Omiya, S	-	0.9–3.4	0.9–1.7	5.5–8.0	6.5–7.5	96.1	*H. litoralis*
56	Fujimi, S	-	0.9–2.6	0.9–1.7	6.5–8.0	6.5–7.5	96.0	*H. litoralis*
62	Higashimatsuyama, S	-	0–3.4	0.9–1.7	6.5–8.0	6.5–7.5	96.0	*H. litoralis*
65	Yachiyo, C	-	0.9–2.6	0.9–1.7	6.5–8.0	6.5–7.5	96.0	*H. litoralis*
83	Nerima, T	Y	0.9–3.4	0.9–1.7	6.5–8.0	6.5–7.5	96.0	*H. litoralis*
89	Fujimi, S	-	0–3.4	0.9–2.6	6.5–8.0	6.5–7.5	96.0	*H. litoralis*
109	Kyowa, I	Y	0–4.3	0.9–2.6	6.5–8.0	6.5–7.5	96.0	*H. litoralis*
120	Toride, I	-	0.9–3.4	0.9–2.6	6.5–8.0	6.5–7.5	96.0	*H. litoralis*
128	Tsurugashima, S	-	0–3.4	0.9–1.7	6.5–8.0	6.5–7.5	96.0	*H. litoralis*
146	Kawagoe, S	-	0–2.6	0–0.9	5.5–8.0	6.5–7.5	96.0	*H. litoralis*
175	Tsurugashima, S	-	0–4.3	0–0.9	6.5–8.0	6.5–7.5	96.0	*H. litoralis*
182	Niiza, S	-	0.9–4.3	0.9–1.7	6.5–8.0	6.5–7.5	96.0	*H. litoralis*
189	Ageo, S	-	0–2.6	0.9–1.7	5.5–8.0	6.5–7.5	96.0	*H. litoralis*
57	Itabashi, T	-	0–3.4	0.9–2.6	6.5–8.0	6.5–7.5	95.9	*H. litoralis*
63	Nerima, T	-	0–3.4	0.9–1.7	6.5–8.0	6.5–7.5	95.9	*H. litoralis*
110	Kyowa, I	Y	0–3.4	0.9–2.6	6.5–8.0	6.5–7.5	95.9	*H. litoralis*
127	Tsurugashima, S	-	0.9–3.4	0.9–1.7	6.5–8.0	6.5–7.5	95.9	*H. litoralis*
187	Koga, I	-	0.9–3.4	0.9–1.7	6.5–8.0	6.5–7.5	95.9	*H. litoralis*
80	Koshigaya, S	-	0–3.4	0–0.9	6.5–8.0	6.5–7.5	95.8	*H. litoralis*
155	Omiya, S	-	0–3.4	0.9–1.7	6.5–8.0	6.5–7.5	95.8	*H. litoralis*
166	Tsurugashima, S	-	0–3.4	0–0.9	6.5–8.0	6.5–7.5	95.8	*H. litoralis*
202	Omiya, S	-	0.9–2.6	0.9–1.7	6.5–8.0	6.5–7.5	95.8	*H. litoralis*
59	Higashichichibu, S	Y	0–3.4	0–0.9	6.5–8.0	6.5–7.5	95.7	*H. litoralis*
79	Shiki, S	Y	0–3.4	0.9–1.7	6.5–8.0	6.5–7.5	95.7	*H. litoralis*
159	Omiya, S	-	0–3.4	0–0.9	6.5–8.0	6.5–7.5	95.7	*H. litoralis*
156	Omiya, S	-	0–3.4	0–0.9	6.5–8.0	6.5–7.5	95.6	*H. litoralis*
178	Urawa, S	-	0–4.3	0.9–1.7	6.5–8.0	6.5–7.5	95.6	*H. litoralis*
165	Warabi, S	-	0–3.4	0–0.9	6.5–8.0	6.5–7.5	95.2	*H. litoralis*
52	Omiya, S	Y	0–3.4	0.9–2.6	6.5–8.0	6.5–7.5	100	*H. trueperi *(AJ310149)
64	Shiki, S	-	0.9–3.4	1.7–2.6	5.5–8.0	6.5–7.5	100	*H. trueperi*
67	Okegawa, S	-	0.9–3.4	1.7–2.6	6.5–8.0	6.5–7.5	100	*H. trueperi*
82	Omiya, S	-	1.7–2.6	1.7–2.6	6.5–8.0	6.5–7.5	100	*H. trueperi*
96	Soka, S	-	0.9–3.4	1.7–2.6	6.5–8.0	6.5–7.5	100	*H. trueperi*
98	Higashimatsuyama, S	Y	0–3.4	0.9–2.6	6.5–8.0	6.5–7.5	100	*H. trueperi*
99	Higashimatsuyama, S	Y	0.9–3.4	0.9–1.7	6.5–8.0	6.5–7.5	100	*H. trueperi*
111	Kyowa, I	-	0–3.4	0.9–1.7	5.5–8.0	6.5–7.5	100	*H. trueperi*
114	Shiki, S	-	0.9–3.4	0.9–1.7	5.5–8.0	6.5–7.5	100	*H. trueperi*
118	Toride, I	Y	0.9–2.6	0.9–1.7	6.5–8.0	6.5–7.5	100	*H. trueperi*
122	Urawa, S	-	0–3.4	0–0.9	6.5–8.0	6.5–7.5	100	*H. trueperi*
150	Tsurugashima, S	Y	0–3.4	0–0.9	6.5–8.0	6.5–7.5	99.8	*H. trueperi*
181	Higashimatsuyama, S	-	0–3.4	0.9–1.7	6.5–8.0	6.5–7.5	99.7	*H. trueperi*
157	Nagareyama, C	-	0–4.3	1.7–2.6	6.5–8.0	6.5–7.5	99.6	*H. trueperi*
58	Kamagaya, C	Y	0–3.4	0–0.9	5.5–8.0	6.5–7.5	99.5	*H. trueperi*
53	Kamagaya, C	Y	0–3.4	0–0.9	5.5–8.0	6.5–7.5	99.4	*H. trueperi*
87	Kawasaki, K	Y	0–3.4	0.9–1.7	6.5–8.0	6.5–7.5	99.3	*H. trueperi*
94	Soka, S	-	0–3.4	0.9–2.6	6.5–8.0	6.5–7.5	99.3	*H. trueperi*
153	Warabi, S	Y	0–3.4	0–0.9	6.5–8.0	6.5–7.5	99.2	*H. trueperi*
163	Kawagoe, S	-	0–3.4	0–0.9	5.5–8.0	6.5–7.5	99.2	*H. trueperi*
142	Kawagoe, S	Y	0–3.4	0.9–1.7	5.5–8.0	6.5–7.5	99.0	*H. trueperi*
140	Urawa, S	Y	0–4.3	0.9–2.6	5.5–8.0	6.5–7.5	98.9	*H. trueperi*
139	Urawa, S	-	0–3.4	0–0.9	6.5–8.0	6.5–7.5	98.6	*H. trueperi*
81	Kasukabe, S	-	0–3.4	0.9–1.7	5.5–8.0	6.5–7.5	98.3	*H. trueperi*
129	Tsurugashima, S	-	0–3.4	0–0.9	6.5–8.0	6.5–7.5	98.3	*H. trueperi*
193	Wako, S	-	0–3.4	0–0.9	6.5–8.0	6.5–7.5	98.1	*H. trueperi*
90	Hatoyama, S	-	1.7–2.6	1.7–2.6	6.5–8.0	6.5–7.5	97.1	*H. trueperi*
158	Nagareyama, C	-	0.9–1.7	0.9–1.7	6.5–8.0	6.5–7.5	96.4	*H. trueperi*
108	Iwatsuki, S	Y	1.7–3.4	1.7–2.6	6.5–8.0	6.5–7.5	100	*L. salicampi *(AY057394)
101	Omiya, S	-	0–3.4	0–0.9	5.5–8.0	6.5–7.5	96.0	*P. ryukyuensis *(AB087828)
174	Higashimatsuyama, S	Y	0–4.3	0–0.9	5.5–8.0	6.5–7.5	96.0	*P. ryukyuensis*
177	Kamifukuoka, S	-	0–3.4	0–0.9	5.5–8.0	6.5–7.5	95.8	*P. ryukyuensis*
198	Kiyose, T	-	0–3.4	0–0.9	5.5–8.0	6.5–7.5	95.6	*P. ryukyuensis*
169	Ageo, S	-	0–4.3	0–0.9	5.5–8.0	6.5–7.5	95.5	*P. ryukyuensis*
121	Toride, I	P	0.9–3.4	0.9–1.7	6.5–8.0	6.5–7.5	93.5	*V. carmonensis *(AJ316302)
141	Ranzan S	-	0–3.4	0.9–1.7	6.5–8.0	6.5–7.5	99.9	*V. halodenitrificans *(AB021186)
148	Sakado, S	-	0–3.4	0–0.9	5.5–8.0	6.5–7.5	99.7	*V. halodenitrificans*
147	Matsubushi, S	-	0–3.4	0.9–1.7	6.5–8.0	6.5–7.5	99.5	*V. halodenitrificans*
160	Fujioka, Tg	-	0–3.4	0–0.9	5.5–8.0	6.5–7.5	94.4	*V. halodenitrificans*
137	Urawa, S	-	0–3.4	0–0.9	6.5–8.0	6.5–7.5	94.1	*V. halodenitrificans*
201	Omiya, S	-	0–3.4	0–0.9	5.5–8.0	6.5–7.5	100	*V. marismortui *(AJ009793)
126	Tsurugashima, S	-	0–3.4	0.9–1.7	6.5–8.0	6.5–7.5	98.3	*V. marismortui*
123	Urawa, S	-	0–3.4	0–0.9	6.5–8.0	6.5–7.5	97.3	*V. marismortui*
161	Tsurugashima, S	P	0–3.4	0–0.9	6.5–8.0	6.5–7.5	96.9	*V. picturae *(AJ315060)
138	Urawa, S	P	0.9–1.7	0.9–1.7	6.5–8.0	6.5–7.5	96.7	*V. picturae*
179	Urawa, S	-	1.7–4.3	1.7–2.6	6.5–8.0	6.5–7.5	95.9	*V. picturae*
149	Ryugasaki, I	-	0.9–2.6	0.9–1.7	6.5–8.0	6.5–7.5	95.8	*V. picturae*
134	Kamifukuoka, S	P	0.9–3.4	0.9–1.7	6.5–8.0	6.5–7.5	95.4	*V. picturae*
135	Urawa, S	P	0–3.4	0.9–1.7	6.5–8.0	6.5–7.5	95.1	*V. picturae*
73	Sakado, S	-	1.7–4.3	1.7–2.6	6.5–8.0	6.5–7.5	88.4	*B. agaradhaerens *(X76445)
								
219	Kasukabe, S	-	0–3.4	0.9–1.7	5.5–8.0	6.5–7.5	100	*B. megaterium *(AY553118)
216	Sekijo, I	-	0–1.7	0–0.9	5.5–8.0	6.5–7.5	99.3	*H. litoralis *(X94558)
214	Sayama, S	-	1.7–2.6	1.7–2.6	5.5–8.0	6.5–7.5	96.3	*H. litoralis*
211	Higashichichibu, S	-	0–3.4	0.9–1.7	5.5–8.0	6.5–7.5	96.1	*H. litoralis*
213	Sekijo, I	-	0–1.7	0–0.9	5.5–8.0	6.5–7.5	95.9	*H. litoralis*
215	Kawagoe, S	-	0–4.3	0.9–1.7	5.5–8.0	6.5–7.5	95.9	*H. litoralis*
212	Sayama, S	-	0–3.4	0.9–1.7	6.5–8.0	6.5–7.5	94.9	*H. litoralis*
218	Toride, I	-	0–4.3	0–0.9	6.5–8.0	6.5–7.5	97.5	*H. trueperi *(AJ310149)
220	Hachioji, T	-	0–3.4	0.9–1.7	5.5–8.0	6.5–7.5	100	*V. halodenitrificans *(AB021186)
217	Itabashi, T	-	0–3.4	0–0.9	5.5–8.0	6.5–7.5	94.5	*V. necropolis *(AJ315056)

Strains No. 1–31 isolated on alkaline medium (pH 9.0), strains No. 51–203 isolated on neutral medium (pH 7.0) and strains No. 211–220 isolated on acidic medium (pH 5.0). Abbreviations of prefectures: C, Chiba; G, Gunma; I, Ibaraki; K, Kanagawa; S, Saitama; T, Tokyo; Tg, Tochigi. Abbreviations of pigmentation:–, None; B, Brown; P, Pink; Y, Yellow. Abbreviations of generic names: *B., Bacillus*; *F., Filobacillus*; *G., Gracilibacillus*; *H., Halobacillus*; *L., Lentibacillus*; *P., Paraliobacillus*; *Pc., Planococcus*; *V., Virgibacillus*.

### Anaerobic halophiles

Five soil samples which gave considerable numbers of colonies on the aerobic cultures were spread on agar plates (pH 7.0) and incubated in anaerobic jar for 3 weeks. No colonies were observed at all, while 30 to 40 colonies appeared from the same soil samples incubated aerobically.

### Growth range of NaCl concentration and pH in liquid media

Of the 27 strains isolated on the alkaline medium, 21 strains did not grow in media without NaCl, and all except one (strain No. 31) showed optimal growth at alkaline pH, 8.5–9.5 in the presence of 10% NaCl. On the other hand, about 78% (116/149 strains) of the strains isolated on neutral and acidic media were shown to be able to grow without added NaCl, and all strains showed optimal growth at pH 6.5–7.5 (Table 1).

Altogether, 176 strains were divided into 3 groups. Strains of group I and group II may be classified as moderately halophilic bacteria, according to the classification proposed by Kushner et al. [6].

Group I (54 isolates) showed optimal growth between 1.7 and 2.6 M NaCl, and no growth in the absence of added NaCl.

Group II (62 isolates) showed optimal growth between 0.9 and 1.7 M NaCl, and growth in the absence of added NaCl.

Group III (60 isolates) showed optimal growth between 0 and 0.9 M NaCl, and growth in the absence of added NaCl.

### Tentative identification of the isolates by partial 16S rRNA gene sequences

Sequences of PCR-amplified partial 16S rRNA genes were determined (about 500 nucleotides), and the 176 strains were tentatively identified by comparing to sequences deposited in databases (Table 1). Summaries of tentative identifications are given in Table 2.

#### Isolates from the alkaline medium

Ten out of 27 strains showed more than 98% sequence similarities to *Bacillus haloalkaliphilus*. It was noteworthy that these isolates differed considerably in the range of NaCl for growth; from 0.9–1.7 M to 1.7–4.3 M. Two isolates possessed 97.2 and 94.0% similarities to *Filobacillus milosensis*, and one isolate was most closely related to *Gracilibacillus halotolerans *(96.1% similarities). Fourteen other isolates had less than 92% sequence similarities to any deposited sequences. Eight isolates showed 86.9–88.0% similarities to '*Bacillus nitritophilus*', and one isolate 87.3% similarity to '*Planococcus psychrotoleratus*' but these species have not been validly published. Similarities of complete sequences of the 14 isolates (data not shown) were less than 92%, thus they may represent novel taxa. Out of these 14 isolates, 5 strains were pigmented brown and 2 isolates were yellow.

**Table 2:** Tentative identification of the isolates by partial 16S rRNA gene sequences.

Tentatively assigned to	pH of isolation media		
	
	5.0	7.0	9.0
*B. haloalkaliphilus *(AJ238041)			10
*B. megaterium *(AY553118)	1		
*F. milosensis *(AJ238042)		17	2
*G. halotolerans *(AB101591)		5	1
*H. karajensis *(AJ486874)		1	
*H. litoralis *(X94558)	6	66	
*H. trueperi *(AJ310149)	1	28	
*L. salicampi *(AY057394)		1	
*P. ryukyuensis *(AB087828)		5	
*V. carmonensis *(AJ316302)		1	
*V. halodenitrificans *(AB021186)	1	5	
*V. marismortui *(AJ009793)		3	
*V. necropolis *(AJ315056)	1		
*V. picturae *(AJ315060)		6	
No closely related species		1	14
Total	10	139	27

Tentatively assigned to	Group I	Group II	Group III

*B. haloalkaliphilus*	9	1	
*B. megaterium*		1	
*F. milosensis*	1	2	16
*G. halotolerans*		1	5
*H. karajensis*		1	
*H. litoralis*	17	38	17
*H. trueperi*	9	10	10
*L. salicampi*	1		
*P. ryukyuensis*			5
*V. carmonensis*	1		
*V. halodenitrificans*		3	3
*V. marismortui*		1	2
*V. necropolis*			1
*V. picturae*	4	1	1
No closely related species	12	3	
Total	54	62	60

Abbreviations of generic names: *B., Bacillus*; *F., Filobacillus*; *G., Gracilibacillus*; *H., Halobacillus*; *L., Lentibacillus*; *P., Paraliobacillus*; *Pc., Planococcus*; *V., Virgibacillus*.

#### Isolates from the neutral medium

Sixty six out of 139 strains showed more than 95% sequence similarities to *Halobacillus litoralis*: 28 isolates possessed more than 98% similarities to *Halobacillus trueperi*: 17 isolates more than 96% similarities to *Filobacillus milosensis*: 6 isolates more than 95% similarities to *Virgibacillus picturae*: 5 isolates more than 94% similarities to *Virgibacillus halodenitrificans*: 3 isolates 97.3, 98.3 and 100% similarities to *Virgibacillus marismortui*: 5 isolates more than 96% similarities to *Gracilibacillus halotolerans*: 5 isolates more than 95% similarities to *Paraliobacillus ryukyuensis*. Three other isolates were most closely related to *Halobacillus karajensis *(94.8%), *Virgibacillus carmonensis *(93.5%) and *Lentibacillus salicampi *(100%), respectively. One isolate had less than 89% similarities to any deposited sequences. In the strains from the neutral medium, 29 strains pigmented yellow, and 5 strains were pigmented pink.

#### Isolates from the acidic medium

Sequences of 6 out of 10 strains were most similar to that of *Halobacillus litoralis*, with more than 94% sequence similarities. Four other strains were most similar to *Virgibacillus necropolis *(94.5%), *Virgibacillus halodenitrificans *(100%), *Halobacillus trueperi *(97.5%) and *Bacillus megaterium *(100%).

### Ratios of halophilic bacteria to total bacteria

The numbers of total bacteria (c.f.u. on plates with no NaCl) and halophilic bacteria were determined in six inland soil samples; three samples had 30–40 colonies on the 20% NaCl agar plates (pH 7.0), and three samples had just 1 colony on the same medium. As shown in Table 1, the number of c.f.u. on the plates ranged from 340 × 1,000 to 28 × 1,000, thus the total bacteria of inland soil samples were in a range from 1.4 × 10^7^/g (340,000 × 20 × 2) to 1.1 × 10^6^/g (28,000 × 20 × 2). Roughly speaking, one tenth of the total bacteria were occupied by endospore-forming bacteria, and only very few of the endospore-forming bacteria, roughly 1 out of 20,000 or more cells, are halophilic bacteria. Table 3(A) also suggests that most, if not all, of halophilic bacteria are surviving as endospores in the soil samples, in a range of less than 1 to about 500/g soil.

**Table 3:** Numbers of colony-forming units of samples of inland soil and seashore sands.

		Numbers of c.f.u.		Numbers of endospore	
			
	Sampling site	NaCl 0%	NaCl 20%	NaCl 0%	NaCl 20%
(A)	Inland soils				
	Warabi, S	340,000	14	89,000	3
	Koto, T	186,000	6	11,000	5
	Toshima, T	209,000	8	27,000	1
	Ina, S	180,000	0	20,000	0
	Shiki, S	113,000	0	13,000	0
	Higashimurayama, T	28,000	0	2,000	0

(B)	Seashore sands				
	Tateyama, C (20 m from sea)	282	6	64	6
	Tateyama, C (20 m from sea)	99	0	17	0
	Tateyama, C (5 m from sea)	501	13	24	4
	Tateyama, C (5 m from sea)	381	7	93	1
	Tateyama, C (0 m from sea)	244	12	8	1
	Tateyama, C (0 m from sea)	289	5	34	4

For the determination of numbers of c.f.u., six inland and six seashore samples (0.5 g) were suspended in distilled water or 10% NaCl solution (2.0 ml), and serially dilutions were spread on agar media (0.1 ml/plate) with no or 20% NaCl, and incubated at 37°C. Numbers of endospore forming bacteria, were determined after heat treatment of the soil suspension at 80°C for 60 min. Numbers are averages of three experiments.

In a separate experiment, 0.5 g of a soil sample was suspended in 2 ml of sterile 10% NaCl, and heated at 80°C. After incubation for 0, 5, 10, 30, and 60 min, three 0.1 ml aliquots were taken, spread on 20% NaCl agar medium (pH 7.0), and incubated for 2 weeks. Data in Table 4 clearly indicated that numbers of c.f.u. (halophilic bacteria) showed little decrease upon heating for 60 min, indicating again that the most of halophilic bacteria were surviving as endospores in soil.

**Table 4:** Numbers of colonies after heat treatment at 80°C for varying time.

	Number of c.f.u			
	
Heat treatment	Plate 1	Plate 2	Plate 3	Average
0 min	10	18	14	14.0
5 min	17	13	13	14.3
10 min	14	11	16	13.7
30 min	19	12	13	14.7
60 min	16	13	14	14.3

The soil sample used was 84-10 (Toride, I).

### Halophilic bacteria in outdoor accumulations?

Outdoor accumulations (dust, fine sands etc.) were collected from places like roofs of buildings, veranda, cars, and barks of trees, where heavy rainfall would wash away the previous ones. Numbers of bacterial cells and endospores present ranged from 0.8 × 10^6 ^to 7.6 × 10^6^/g and from 89 × 10^3 ^to 812 × 10^3^/g, respectively. No colonies, however, were observed on any 20% NaCl plates (pH 5.0, 7.0, and 9.0) from 0.5 g samples, even after 8 weeks incubation. Repeated incubations of outdoor accumulations collected 2 weeks after rainfall gave no colonies at all.

### Halophilic bacteria in seashore soil (sands)

Six samples were collected from three spots of seashore in Tateyama of Chiba prefecture, a city confronting Tokyo Bay (Fig. 1). Samples were suspended, heated at 80°C, and subjected to colony counting. Table 3(B) showed clearly that the total numbers of bacteria and endospores were roughly 1000 time smaller than those of inland soil samples. Numbers of halophilic bacteria, however, were almost the same as those of inland soil samples.

### NaCl contents of samples

Analyses of Cl content of soil samples suggested that NaCl contents of soil samples taken from near seashore were as high as 15–20 mg NaCl/g, whereas the 360 inland soil samples contained less than 1 mg NaCl/g.

### Haloarchaea in soil samples?

The agar plate with 20% NaCl used in this study was based on the medium No. 168 recommended by JCM (Japan Collection of Microorganisms) for the cultivation of haloarchaea. All of the pink to brown colonies that might be haloarchaea were picked up, but they all belonged of the family *Bacillaceae*. To ascertain that haloarchaea are not present in the soil samples, at least to the limit of detection, soil samples were spread on agar plates of 20% NaCl, pH 7.0 and 9.0, supplemented with 30 μg/ml ampicillin. Generally, numbers of colonies decreased to less than one tenth of those obtained on plates without ampicillin. From five soil samples out of 107 samples tested, four colonies were obtained on plates of pH 7.0, and 12 colonies on those of pH 9.0. No colony appeared from seven samples from seashore at Tateyama. The colonies were transferred to liquid media containing ampicillin, and 10 strains that grew were subjected DNA extraction and PCR amplification using both bacterial and archaeal 16S rRNA gene primer sets. All of them yielded amplification bands only with bacterial primers, suggesting they were not haloarchaea but halophilic bacteria harboring plasmids with *β*-lactamase genes.

## Discussion

Definition of "moderate halophiles", "extreme halophiles" and "halotolerant" has long been given by Larsen [18] and Kushner & Kamekura [6]. In this paper, we defined "halophilic bacteria", for convenience, as microorganisms able to form colonies on agar plates containing 20% (3.4 M) added NaCl. Strictly speaking, there existed moderate halophiles that were not able to grow in the presence of 20% NaCl.

### Is the distribution of halophilic Bacteria and Archaea restricted?

Since halophilic bacteria have been recognized to live in the Dead Sea [18], numerous halophilic and halotolerant microorganisms, both aerobic and anaerobic, both Bacteria and Archaea, have been isolated from saline environments. Thanks to the enthusiastic devotion of A. Oren on halophilic microorganisms [7], we know that halophilic Bacteria are not restricted to the class Bacilli (*Bacillus sensu lato*) but distributed through classes of Cyanobacteria, *α*-, *β*-, *γ*-, and *δ*-proteobacteria, Clostridia, Actinobacteria, Flavobacteria, etc. Although Oren defined the "halophilic" as tolerance to 10% (100 g/L) NaCl in his book [7], some of the halophilic microorganisms are able to grow in the presence of 20% NaCl. We also know that all microorganisms that were intentionally isolated as halophiles are inhabitants of saline environments. On the other hand, some bacteria isolated from soil are able to tolerate high NaCl concentrations. For example, *Bacillus clarkii*, *B. agaradhaerens*, and *B. pseudofirmus *are tolerant up to 16% or 17% NaCl [13]. To the best knowledge of the authors of this paper, no reports have been published on the isolation of microorganisms able to grow at 20% or higher NaCl concentrations from ordinary non-saline soil samples. It has tacitly been believed that habitats of halophiles able to grow in media containing more than 20% are restricted to saline environments [14,15].

### Halophilic bacteria are isolated from soil samples

In the present study, we have demonstrated that halophilic bacteria that are able to grow in the presence of 20% NaCl are inhabiting almost everywhere in non-saline environments such as ordinary garden soils, yards, fields and roadways in an area surrounding Tokyo. We isolated 176 strains, and analysis of partial sequences of their 16S rRNA genes showed that some of them possessed similarities higher than 94.8% with those of *Bacillus haloalkaliphilus *[20], *Filobacillus milosensis *[21], *Gracilibacillus halotolerans *[22], *Halobacillus karajensis *[23], *Halobacillus litoralis *[24], *Halobacillus trueperi *[24], *Lentibacillus salicampi *[25], *Paraliobacillus ryukyuensis *[26], *Virgibacillus halodenitrificans *[27], *Virgibacillus marismortui *[28] and *Virgibacillus picturae *[29]. Most of the strains of these species have been isolated from saline environments, and were reported to be halophilic, capable of growth at 20% NaCl. All strains of species of the genera of the family Bacillacea were endospore formers, except *Bacillus saliphilus *[30]. Sequences of 15 isolates (14 isolates from alkaline medium, and one isolated from neutral medium) showed similarities less than 92% to any deposited sequences, thus they may represent novel taxa within the family *Bacillaceae*. For unknown reason(s), cells of some colonies on the initial isolation plates failed to grow when transferred to fresh plates, but grew on plates with lower NaCl concentrations. Some growth factors present in soil might be responsible for this phenomenon [31].

A large number of halophilic bacteria of group I (no grow without added NaCl) were shown to be alkaliphilic, and most of the group II and III, which grew without added NaCl, were neutrophilic. The haloalkaliphiles, halophilic and alkaliphilic bacteria [2], have been found mainly in extremely alkaline and saline environments, which were distributed in the Rift Valley lakes of East Africa, soda lakes of the United States and Inner Mongolia of China, etc.

Quite interesting is the fact that none of the isolates of halophilic bacteria showed similarities with any halophilic microorganisms of the classes mentioned above other than the endospore-forming *Bacillus sensu lato*. There remains a possibility, however, that colonies of halophilic bacteria other than Bacilli on agar plates unfortunately escaped from being picked up for purification. Another possibility is that those halophilic bacteria lost the ability to form colonies on the particular agar plates we used during repeated transfers in the purification procedures, or that they simply did not form colonies because of unsuitableness of the composition of agar plates to them.

### Are the halophilic Bacilli indigenous to soil?

Spore-formers of the family *Bacillaceae *were easily isolated from a number of environments by suspending a sample in water and heating at 80°C for 10 to 30 min, even from environments unrelated to their growth requirements. For example, a thermophile *Geobacillus stearothermophilus *was isolated from ordinary soil, and many of the alkaliphilic Bacillus species have been isolated from soils that were not particularly alkaline [32]. Now we know that roughly one tenth of the culturable total bacteria present in ordinary soil were endospore-forming bacteria, only very few of them were halophilic bacteria, and most of the halophilic bacteria were surviving as endospores. Also, the exact distribution of so called "soil microorganisms" of each taxon may fluctuate depending on the soil and also on season, number of cells of alkaliphiles and methanogens have been shown to be an order of 10^4 ^to 10^5^/g of neutral soil [2,4]. The numbers of endospores of the halophilic bacteria ranged from less than 1 to 500/g in our experiments. A question is "Are the endospores of halophilic bacteria indigenous to soil, and if not, where did they come from?" Here we remember that (i) NaCl contents of soil samples taken from near seashore contained as high as 15–20 mg NaCl/g, whereas the 360 inland soil samples contained less than 1 mg NaCl/g, and that (ii) numbers of endospores of halophilic bacteria were almost the same in the inland soil samples and the seashore sand samples. These facts strongly suggest that the endospores of halophilic bacteria are neither from the sea nor from minute highly saline niche produced by evaporation of seawater on seashore. Then, where are they from?

### Bacteria in Asian Dust?

Although we have no confirming data at present, we may speculate that the halophilic bacteria have been transported by westerlies either as vegetative cells or as endospores from the indigenous highly saline environments, such as salt lakes in Inner Mongolia or salterns confronting Yellow Sea or East Sea (Japan Sea) in Korea. In fact, several novel genera and species have been isolated from these areas recently. It has been realized that Aeolian dust (mineral dust) and sand storms are plaguing North-East Asia [33]. The storms originate in the arid inland parts of China and Mongolia and blow across the Korean peninsula and Japan. It is believed that cold air masses from Siberia whip deserts and soils eastward after the dry continental winter. The dusts kicked up into the jet stream are carried by the prevailing westerlies across mainland Asia, over the Sea of Japan and Pacific Ocean and reach into the main land United States in just five days. It is demonstrated that the dusts reach even to Hawaii, which is over 6,000 km away [34,35]. China's sand storms are referred to as Huangsha in China, "Asian dust" or Whangsa in Korea, and "Yellow sand" or Kosa event in Japan. The authors of this study believe that bacteria, at least their endospores, thriving in salt lakes, soda lakes, and the surrounding saline soils in the arid region have been kept carried to Japan for thousands of years together with the mineral dusts. A long-distance transport of fungal spores by winds across the English Channel was demonstrated by Hirst et al. [36]

Cells of non-endospore-forming halophilic bacteria and halophilic archaea (see below) thriving in the saline environments would be flown to Japan by westerlies together with the endospores, but they would die sooner or later after they arrive at soil, at least because of the hypotonic conditions caused by rainfall.

### How long have they thrived in soil?

If the above speculation is correct, the next question is "At what density are there the endospores of halophilic bacteria in the yellow sand and how long do they survive in soil?" The fact that dusts accumulated for several days after the last rainfall gave no colonies on 20% NaCl agar plates suggests that frequencies of the presence of endospores of halophilic bacteria transported by the westerlies are very low, and 40 colonies that appeared on the agar plate are the result of precipitation of the endospores for a very long time span. Endospores are known to be able to survive for quite a long time, at least several decades, or even thousands of years. There is a famous case that a endospore of bacteria closely related to extant *Bacillus sphaericus *was revived and cultured from the abdominal contents of extinct bees preserved for 25 to 40 million years in buried Dominican amber [37]. An even more spectacular claim was made that a bacteria closely related to *Virgibacillus marismortui *was isolated from fluid inclusions in rock salt crystals of Permian age, over 250 million years old [38]. Although there is no well-documented data on the longevity of endospores in the soil, we may expect they can survive at least for decades.

### Halophilic strains of the class Bacilli from non-saline environments

Recently, several moderately halophilic bacteria, representing novel species of the genus *Halobacillus *(97.1 to 98.4% similarities to *Halobacillus litoralis *in the 16S rRNA gene sequences) were isolated by enrichment culture in a medium containing 20% NaCl from damaged medieval wall paintings and building materials in Austria [39]. To the best knowledge of authors of this paper, this is the only exception of the isolation of halophilic bacteria from non-saline environments. *Virgibacillus carmonensis*, *V. necropolis *and *V. picturae *isolated from samples of biofilm formation on the mural paintings in Spain [28] are moderately halophilic with optimal growth at NaCl concentration of 5 to 10%, but it is not clear if they are able to grow at 20% NaCl.

## Conclusion

An answer to a question "Are the endospores of halophilic bacteria indigenous to soil, and if not, where did they come from?" will be that the endospores of halophilic bacteria are NOT indigenous to soil, and they are neither from sea nor from minute highly saline niche produced by evaporation of seawater on seashore. We may speculate that the endospores of halophilic bacteria detected in this study were the results of precipitation in long-term, for years at least, from atmosphere that have been transported by westerlies from the indigenous highly saline environments, such as salt lakes in Inner Mongolia or salterns in Korea.

## Methods

### Samples of soils, accumulations on roofs etc., and seashore sands

A total of 360 soil samples were collected in an area (103 km by 126 km) surrounding Tokyo, Japan (Tokyo, Saitama-, Chiba-, Kanagawa- and Ibaraki- prefecture) (Fig. 1). Each soil sample was taken from surfaces such as gardens, fields, yards and roadways, which was separated each other by at least 1 km. There exist no highly saline environments in this region such as salterns and salt lakes. On the other hands, 6 accumulation samples were taken from roof of a car, roofs of three buildings, and veranda of two rooms of building in the campus of Toyo University or nearby cities. Several samples were also taken from seashore of Tateyama, a city southern part of Chiba-prefecture confronting Tokyo-Bay (Fig. 1). Each soil sample was collected into 50 ml sterile FALCON tubes (Becton Dickinson) with sterile spatula, and kept at room temperature until use.

### Agar media for the isolation of halophilic bacterial and archaeal strains

A complex growth medium contained the following ingredients (per liter). 5.0 g casamino acids (Difco), 5.0 g yeast extract (Difco), 1.0 g sodium glutamate·H_2_O, 3.0 g trisodium citrate·2H_2_O, 2.0 g KCl, 0.2 g MgSO_4_·7H_2_O, 36 mg FeCl_2_·4H_2_O, 200 g (3.4 M) NaCl and 20 g Bacto-agar (Difco), pH 7.2. After autoclaving, pH was adjusted to 5.0, 7.0 or 9.0 by adding pre-calculated amounts of diluted sterile H_2_SO_4 _or Na_2_CO_3 _solutions. Soil samples (0.5 g each) were placed directly on the three agar plates of different pH, spread with spatula, and incubated at 37°C for 3 weeks in plastic bags to prevent desiccation. Colonies were picked up, transferred to fresh agar plates of the same pH, and pure cultures were obtained by plating serial dilutions and repeated transfers on agar plates of the same medium. In some experiments, the agar plates of pH 7.0 and 9.0 were supplemented with 30 μg/ml ampicillin for the selective isolation of haloarchaeal strains.

### Determination of growth range

Growth was determined by inoculating pre-cultures of purified strains into 30 ml test tubes each containing 3 ml liquid media with varying NaCl concentrations (0, 0.9, 1.7, 2.6, 3.4, 4.3 and 5.2 M, pH 7 or 9) and pH (5.0, 5.5, 6.0, 6.5, 7.0, 7.5, 8.0, 8.5, 9.0, 9.5 and 10.0, in the presence of 1.7 M NaCl) and shaken at 37°C with 120 rpm. Growth was monitored by taking 0.1 ml culture broth periodically and measuring absorbance at 660 nm.

### Measurements of numbers of total bacterial cells, halophilic bacteria, and endospore-forming bacteria

For the estimation of the numbers of colony forming unit and halophilic bacteria present in the soil samples, 0.5 g samples were suspended in 2 ml of distilled water or sterile 10% NaCl solution, diluted serially with the same solution, and 0.1 ml each was spread on agar plates without added NaCl or of 20% NaCl, respectively, pH 7.0, followed by incubation at 37°C for 2 weeks. Separate experiments on 9 soil samples have shown that increasing concentrations of NaCl of agar plates for colony counting (0, 5, 10, 15 and 20%) just decreased the number of c.f.u. The numbers of total endospore-forming bacteria were determined after heat treatment of the soil suspension at 80°C for 60 min, followed by dilution and spreading on the plates without added NaCl. Heat resistance of endospores is known to differ in different strains of *Bacillaceae *and depends on the NaCl concentration during heating [40,41].

### Spore-staining

Endospore formations of the isolates were examined with a phase-contrast microscope (×1,000) or after spore-staining according to the method of Wirtz-Conklin [17].

### Anaerobic growth

For the detection of anaerobic halophilic bacteria, 5 soil samples were spread on the agar plates as described above, and incubated at 37°C in an anaerobic jar using a deoxygenation reagent, Anaero Pauch Anaero, and Anaero Pack Rectangular Jar (Mitsubishi Gas Chemical Co., Inc., Tokyo).

### Phylogenetic analysis

Total DNAs were extracted by the method of Cline *et al*. [42]. The 16S rRNA genes were amplified by PCR and directly sequenced by using the ABI PRISM^® ^BigDye Terminator v3.1 Cycle Sequencing Kits (Applied Biosystems) with the following forward and reverse primers for Bacteria: 5'-AGAGTTTGATCCTGGCTCAG-3' (positions 8–27 according to *Escherichia coli *numbering) and 5'-GACTACCAGGGTATCTAATC-3' (positions 805–786) on the ABI PRISM^® ^310 Genetic Analyzer (Applied Biosystems). In some experiments, the complete 16S rRNA genes were amplified by PCR with the forward primer and a reverse primer: 5'-GGCTACCTTGTTACGACTT-3' (positions 1510–1492). Archaeal primer sets were 5'-ATTCCGGTTGATCCTGCCGG-3' (positions 6–25 according to *E. coli *numbering) and 5'-AGGAGGTGATCCAGCCGCAG-3' (positions 1540–1521). The closest known relatives of the sequenced organisms were determined by sequence database searches. These sequences and those of known related strains retrieved from the DNA Data Bank of Japan [43-45] were aligned using the CLUSTAL W Multiple Sequence Alignment Program [46]. The phylogenetic tree was reconstructed by the neighbour-joining method [47] and was evaluated by bootstrap sampling [48].

### Estimation of NaCl contents, pH, and water content of soil samples

Ten grams of each soil sample was suspended in 100 ml of distilled water, shaken for 1 h, and supernatants were obtained by centrifugation. NaCl content in soil samples was calculated by determining Cl content of the soil extract by the method of Mohr [49]. The pH of the soil extract was determined with a pH meter. Water content was determined by measuring the decrease of weight after heating 1 g soil sample at 120°C for 1, 2 and 3 hours.

## Authors' contributions

AE carried out isolation of strains and characterization, sequencing of 16S rRNA genes, analyses, study design, and drafted the manuscript.

MH carried out isolation of strains and characterization.

TF participated in the design of study.

TM participated in the design of study.

MK participated in the study design and drafted the manuscript.

RU directed the research and drafted the manuscript.

All authors have read and approved the final manuscript.

## References

[B1] Horikoshi K Grant WD eds Extremophiles – microbial life in extreme environments 1998 New York, Wiley-Liss

[B2] Horikoshi K Alkaliphiles: some applications of their products for biotechnology Microbiol Mol Biol Rev 1999 63 735 750 10585964 10.1128/mmbr.63.4.735-750.1999 PMC98975

[B3] Sneath PHA Endospore-forming Gram-positive rods and cocci Bergey's Manual of Systematic Bacteriology 1986 2 Baltimore, Williams & Wilkins 1104 1207

[B4] Mayer HP Conrad R Factors influencing the populations of methanogenic bacteria and the initiation of methane production upon flooding of paddy soil FEMS Microbiol Ecol 1990 73 103 111 10.1016/0378-1097(90)90656-B

[B5] Peters V Conrad R Methanogenic and other strictly anaerobic bacteria in desert soil and other oxic soils Appl Environ Microbiol 1995 61 1673 1676 16535011 10.1128/aem.61.4.1673-1676.1995 PMC1388429

[B6] Kushner DJ Kamekura M Physiology of halophilic eubacteria Halophilic bacteria 1988 I Boca Raton, CRC Press 109 140

[B7] Oren A Halophilic Microorganisms and their environments 2002 Dordrecht, Kluwer Academic

[B8] Gonzalez C Gutierrez C Ramirez C *Halobacterium vallismortis *sp. nov. an amylolytic and carbohydrate-metabolizing, extremely halophilic bacterium Can J Microbiol 1978 24 710 715 667737 10.1139/m78-119

[B9] Zvyagintseva IS Tarasov AL Extreme halophilic bacteria from saline soils Mikrobiologiya 1987 56 839 844

[B10] Bouchotroch S Quesada E del Moral A Llamas I Bejar V *Halomonas maura *sp. nov., a novel moderately halophilic, exopolysaccharide-producing bacterium Int J Syst Evol Microbiol 2001 51 1625 32 11594589 10.1099/00207713-51-5-1625

[B11] Hao MV Kocur M Komagata K *Marinococcus *gen nov., a new genus for motile cocci with meso-diaminopimelic acid in the cell wall; and *Marinococcus albus *sp. nov. and *Marinococcus halophilus *(Novitsky and Kushner) comb. nov J Gen Appl Microbiol 1984 30 449 459

[B12] Yoon JH Kang KH Park YH *Halobacillus salinus *sp. nov., isolated from a salt lake on the coast of the East Sea in Korea Int J Syst Evol Microbiol 2003 53 687 93 10.1099/ijs.0.02421-0 12807188

[B13] Nielsen P Fritze D Priest FG Phenetic diversity of alkaliphilic *Bacillus *strains: proposal for nine new species Microbiology 1995 141 1745 1761

[B14] Onishi H Fuchi H Konomi K Hidaka O Kamekura M Isolation and distribution of a variety of halophilic bacteria and their classification by salt-response Agric Biol Chem 1980 44 1253 1258

[B15] Saiz-Jimenez C Laiz L Occurrence of halotolerant/halophilic bacterial communities in deteriorated monuments Int Biodeterior Biodegrad 2000 46 319 326 10.1016/S0964-8305(00)00104-9

[B16] Kamekura M Dyall-Smith ML Taxonomy of the family Halobacteriaceae and the description of two new genera *Halorubrobacterium *and *Natrialba* J Gen Appl Microbiol 1995 41 333 350

[B17] Murray PR Baron EJ Pfaller MA TeNover FC Yolken RH Manual of Clinical Microbiology 1999 7 Washington, D.C., ASM Press

[B18] Larsen H Halophilism The Bacteria 1962 4 297 342

[B19] Wilkansky B Life in the Dead Sea Nature 1936 138 467

[B20] Fritze D *Bacillus haloalkaliphilus *sp. nov Int J Syst Bacteriol 1996 46 98 101

[B21] Schlesner H Lawson PA Collins MD Weiss N Wehmeyer U Volker H Thomm M *Filobacillus milensis *gen. nov., sp. nov., a new halophilic spore-forming bacterium with Orn-D-Glu-type peptidoglycan Int J Syst Evol Microbiol 2001 51 425 431 11324591 10.1099/00207713-51-2-425

[B22] Waino M Tindall BJ Schumann P Ingvorsen K *Gracilibacillus *gen. nov., with description of *Gracilibacillus halotolerans *gen. nov., sp. nov.; transfer of *Bacillus dipsosauri *to *Gracilibacillus dipsosauri *comb. nov., and *Bacillus salexigens *to the genus *Salibacillus *gen. nov., as *Salibacillus salexigens *comb. nov Int J Syst Bacteriol 1999 49 821 831 10319508 10.1099/00207713-49-2-821

[B23] Amoozegar MA Malekzadeh F Malik KA Schumann P Sproer C *Halobacillus karajensis *sp. nov., a novel moderate halophile Int J Syst Evol Microbiol 2003 53 1059 1063 10.1099/ijs.0.02448-0 12892126

[B24] Spring S Ludwig W Marquez MC Ventosa A Schleifer KH *Halobacillus *gen. nov., with descriptions of *Halobacillus litoralis *sp. nov., and *Halobacillus trueperi *sp. nov., and transfer of *Sporosarcina halophila *to *Halobacillus halophilus *comb. nov Int J Syst Bacteriol 1996 46 492 496

[B25] Yoon JH Kang KH Park YH *Lentibacillus salicampi *gen. nov., sp. nov., a moderately halophilic bacterium isolated from a salt field in Korea Int J Syst Evol Microbiol 2002 52 2043 2048 10.1099/ijs.0.02335-0 12508866

[B26] Ishikawa M Ishizaki S Yamamoto Y Yamasato K *Paraliobacillus ryukyuensis *gen. nov., sp. nov., a new Gram-positive, slightly halophilic, extremely halotolerant, facultative anaerobe isolated from a decomposing marine alga J Gen Appl Microbiol 2002 48 269 279 12501437 10.2323/jgam.48.269

[B27] Yoon JH Oh TK Park YH Transfer of *Bacillus halodenitrificans *Denariaz et al. 1989 to the genus *Virgibacillus *as *Virgibacillus denitrificans *comb. nov Int J Syst Evol Microbiol 2004 54 2163 2167 10.1099/ijs.0.63196-0 15545452

[B28] Arahal DR Marquez MC Volcani BE Schleifer KH Ventosa A *Bacillus marismortui *sp. nov., a new moderately halophilic species from the Dead Sea Int J Syst Bacteriol 1999 49 521 530 10319473 10.1099/00207713-49-2-521

[B29] Heyrman J Logan NA Busse HJ Balcaen A Lebbe L Rodriguez-Diaz M Swings J De Vos P *Virgibacillus carmonensis *sp. nov., *Virgibacillus necropolis *sp. nov. and *Virgibacillus picturae *sp. nov., three novel species isolated from deteriorated mural paintings, transfer of the species of the genus *Salibacillus *to *Virgibacillus*, as *Virgibacillus marismortui *comb. nov. and *Virgibacillus salexigens *comb. nov., and emended description of the genus *Virgibacillus* Int J Syst Evol Microbiol 2003 53 501 511 10.1099/ijs.0.02371-0 12710619

[B30] Romano I Lama L Nicolaus B Gambacorta A Giordanao A *Bacillus saliphilus *sp. nov., isolated from a mineral pool in Campania, Italy Int J Syst Evol Microbiol 2005 55 159 163 10.1099/ijs.0.63298-0 15653870

[B31] Aagot N Nybroe O Nielsen P Johnsen K An altered *Pseudomonas *diversity is recovered from soil by using nutrient-poor *Pseudomonas*-selective soil extract media Appl Environ Microbiol 2001 67 5233 5239 10.1128/AEM.67.11.5233-5239.2001 11679350 PMC93295

[B32] Krulwich TA Guffani AA Alkalophilic bacteria Annu Rev Microbiol 1989 43 435 463 10.1146/annurev.mi.43.100189.002251 2679360

[B33] North-East Asian dust and sand storms growing in scale and intensity; UNEP warns of 'The Globalization of Environmental Problems' http://www.un.org/News/Press/docs/2004/unep216.doc.htm

[B34] Duce RA Unni CK Ray BJ Prospero JM Merrill JT Long-range atmospheric transport of soil dust from Asia to the tropical north Pacific: temporal variability Science 1980 209 1522 1524 17745962 10.1126/science.209.4464.1522

[B35] Chadwick OA Derry LA Vitousek PM Huebert BJ Hedin LO Changing sources of nutrients during four million years of ecosystem development Nature 1999 397 491 497 10.1038/17276

[B36] Hirst JM Stedman OJ Hurst GW Long-distance spore transport: Vertical sections of spore clouds over the sea J Gen Microbiol 1957 48 357 377 10.1099/00221287-48-3-357 6052629

[B37] Cano RJ Borucki MK Revival and identification of bacterial spores in 25- to 40-million-year-old Dominican amber Science 1995 268 1060 1064 7538699 10.1126/science.7538699

[B38] Vreeland RH Rosenzweig WD Powers DW Isolation of a 250 million-year-old halotolerant bacterium from a primary salt crystal Nature 2000 407 8 10.1038/35038060 11057666

[B39] Piñar G Ramos C Rölleke S Schabereiter-Gurtner C Vybiral D Lubiz W Denner EBM Detection of indigenous *Halobacillus *populations in damaged ancient wall paintings and building materials: molecular monitoring and cultivation Appl Environ Microbiol 2001 67 4891 4895 10.1128/AEM.67.10.4891-4895.2001 11571198 PMC93245

[B40] Roberts TA Hitchins AD Gould GW, Hurst A Resistance of spores The Bacterial Spore 1969 London, Academic Press 611 670

[B41] Murrell WG Warth AD Campbell LL, Halvorson HO Composition and heat resistance of bacterial spores Spores III 1965 Ann Arbor, American Society for Microbiology 1 24

[B42] Cline SW Schalkwyk LC Doolittle WF Transformation of the archaebacterium *Halobacterium volcanii *with genomic DNA J Bacteriol 1989 171 4987 4991 2768194 10.1128/jb.171.9.4987-4991.1989 PMC210307

[B43] Miyazaki S Sugawara H Gojobori T Tateno Y DNA Data Bank of Japan (DDBJ) in XML Nucl Acids Res 2003 30 13 16 10.1093/nar/gkg088 PMC165535 12519938

[B44] Pearson WR Lipman DJ Improved tools for biological sequence comparison Proc Natl Acad Sci USA 1988 85 2444 2448 3162770 10.1073/pnas.85.8.2444 PMC280013

[B45] Lipman DJ Pearson WR Rapid and sensitive protein similarity searches Science 1985 227 1435 1441 2983426 10.1126/science.2983426

[B46] Thompson JD Higgins DG Gibson TJ CLUSTAL W: Improving the sensitivity of progressive multiple sequence alignment through sequence weighting, position-specific gap penalties and weight matrix choice Nucl Acids Res 1994 22 4673 4680 7984417 10.1093/nar/22.22.4673 PMC308517

[B47] Saitou N Nei M The neighbour-joining methods: a new method for reconstructing phylogenetic trees Mol Biol Evol 1987 4 406 425 3447015 10.1093/oxfordjournals.molbev.a040454

[B48] Felsenstein J Confidence limits on phylogenies: an approach using the bootstrap Evolution 1985 39 783 791 28561359 10.1111/j.1558-5646.1985.tb00420.x

[B49] American Public Health Association Standard methods for the examination of water and wastewater 1998 20 American Public Health Association, Washington, D.C

